# PW02-027 - CAPS and cost-effectiveness analysis project

**DOI:** 10.1186/1546-0096-11-S1-A168

**Published:** 2013-11-08

**Authors:** O Della Casa Alberighi, L Accame, J Frenkel, M Gattorno, A Martini, B Neven, P Quartier, F Pierotti, G Turchetti

**Affiliations:** 1 Clinical Pharmacology and Clinical Trial Unit, Giannina Gaslini Institute Pediatric Hospital, Genova, Italy; 2 Pediatric Rheumatology, University Medical Center Utrecht, Utrecht, The Netherlands; 3 2nd Division of Pediatrics, Giannina Gaslini Institute Pediatric Hospital, Genova, Italy; 4 Pediatric Immunology, Haematology and Rheumatology Unit , Assistance Publique-Hopitaux de Paris Hopital Necker, Paris, France; 5 Health Economy, Scuola Superiore S. Anna, Pisa, Italy

## Introduction

Ultra-orphan drugs are medicines used to treat exceptionally rare diseases that are chronically debilitating or life-threatening. Low patient numbers make it difficult for pharmaceutical companies to recoup research and development costs, and consequently these medicines are generally very expensive on a per patient basis. European Union (EU) regulations promote the development of orphan drugs; but to contain costs, EU healthcare systems will increasily need the cost-effectiveness analysis (CEA) of therapies when deciding if they should be funded. Conventional methods for CEA of drugs for common conditions do not apply to ultra-orphan drugs; therefore, additional factors need to be considered.

## Objectives

Using the case of ultra-orphan cryopyrin associated periodic syndromes (CAPS) currently investigated by the EuroFever registry, the RaDiCEA (Rare Diseases & Cost-Effectiveness Analysis) project is aimed at collecting prospective efficacy, safety, tolerability, treatment adherence (effectiveness data), cost of illness (COI) information, and relative effectiveness of life-long treatment strategies, and at elaborating on CEA modeling in ultra rare diseases.

## Methods

### Design and setting

As a EuroFever registry spin-off, a three-year, international, multicentre, observational, cost-effectiveness study will be conducted in approx. 150 CAPS patients through the Paediatric Rheumatology INternational Trials Organisation (PRINTO) network.

### Participants

The EuroFever registry project (http://www.printo.it/eurofever/) involves so far 170 centres of Paediatric Rheumatology and centres of reference for all autoinflammatory diseases in 45 Countries worldwide.

## Results

### Main outcome measures

They will be the retention on treatment and reasons of treatment withdrawal for effectiveness. For safety, the incidence rates of anti-IL-1 agents-emergent adverse events (AEs) and serious AEs will be evaluated in comparison with incidence rates observed in CAPS subjects not exposed to anti-IL-1 agents. The bases for a cost-effectiveness model in CAPS will be set by means of a COI evaluation, and of a comparative economic evaluation of different treatment strategies in the National Health Systems’(NHS) perspectives, using CEA of direct health costs (Incremental Cost Effectiveness Ratio - ICER), and by measuring quality adjusted life years (QALY), and organ/system damage prevention up to three years.

### Expected results

The RaDiCEA project will assess the long-term effectiveness of different potentially life-long treatment strategies and COI, while exploring the feasibility of a new CEA model to be generated from a rare disease (CAPS) registry.

## Conclusion

As expensive medications like “biologicals” show promising results in some patients with “ultra-orphan” diseases, it becomes more and more important to have detailed information on as many patients as possible. A promising new international collaboration aims to develop a model to evaluate both costs and (long-term) benefits in an ultra-orphan group of diseases known as CAPS. The same model may be used in other very rare disorders.

## Disclosure of interest

None declared.

